# Applications and Advantages of Cellulose–Chitosan Biocomposites: Sustainable Alternatives for Reducing Plastic Dependency

**DOI:** 10.3390/polym17010023

**Published:** 2024-12-26

**Authors:** Akmaral Darmenbayeva, Gaziza Zhussipnazarova, Reshmy Rajasekharan, Bakytgul Massalimova, Roza Zharlykapova, Aisha Nurlybayeva, Zhazira Mukazhanova, Gulsim Aubakirova, Bahyt Begenova, Saltanat Manapova, Kamila Bulekbayeva, Assem Shinibekova

**Affiliations:** 1 Department of Chemistry and Chemical Technology, M.Kh. Dulaty Taraz University, Taraz 080000, Kazakhstan; ros61_2010@mail.ru (R.Z.); rustem_ergali@mail.ru (A.N.); nurhat2000@mail.ru (K.B.); aa.shinibekova@dulaty.kz (A.S.); 2 Department of Science and Humanities, Providence College of Engineering, Ala 689122, Kerala, India; reshmypkumar@gmail.com; 3 Department of Chemistry and Chemical Engineering, M. Kozybayev North Kazakhstan University, Petropavlovsk 150000, Kazakhstan; bkmasalimova@ku.edu.kz (B.M.); gbaubakirova@ku.edu.kz (G.A.); bebegenova@ku.edu.kz (B.B.); 4 Higher School of IT and Natural Sciences, S. Amanzholov East Kazakhstan University, Ust-Kamenogorsk 070010, Kazakhstan; mukazhanovazhb@mail.ru; 5 Department of Chemistry, S. Amanzholov East Kazakhstan University, Ust-Kamenogorsk 070010, Kazakhstan; salta_kz_18@mail.ru

**Keywords:** cellulose–chitosan biocomposites, biodegradable materials, eco-friendly packaging, drug delivery, wastewater treatment, sustainable materials

## Abstract

This review presents a comprehensive review of cellulose–chitosan-based biocomposites that have high potential as sustainable alternatives to synthetic polymers. These biocomposites, due to biocompatibility, biodegradability, and antimicrobial properties, attract attention for wide application in various industries. This review includes modern methods for producing cellulose–chitosan composites aimed at improving their mechanical and chemical properties, such as strength, flexibility, and water resistance. Particular attention is paid to the use of composites in packaging materials, where they provide protection and durability of products, and help reduce the environmental footprint. In medicine, such composites are used for drug delivery and tissue engineering, providing controlled release of active substances and tissue regeneration. In addition, their advantages in wastewater treatment are discussed, where the composites effectively remove heavy metal ions and organic pollutants due to their high sorption capacity. This study focuses on the wide potential of cellulose–chitosan biocomposites and their role in solving environmental problems.

## 1. Introduction

Nowadays, the excessive use of plastics, coupled with insufficient recycling efforts, has caused significant environmental damage [1]. One potential biomaterial for replacing plastics is cellulose, which is the most abundant natural biomaterial [2]. Cellulose possesses a range of favorable properties, such as widespread availability, cost-effectiveness, biocompatibility, low toxicity, lightweight, effective oxygen gas barrier properties, excellent mechanical and optical properties, surface tailoring, high stiffness, good elasticity, flexibility, and water-holding capacity. Consequently, cellulose is an attractive biomaterial for replacing non-degradable petroleum-based materials in various fields, including food packaging, medicine, the textile industry, energy, and sensing applications, as well as in wastewater remediation [3,4,5,6].

However, the application of cellulose-based biomaterials is limited due to their lack of antibacterial properties. To address this challenge, cellulose is combined with chitosan, an environmentally benign material derived from chitin—the second most abundant and widespread organic compound. Chitosan is a promising material to replace plastics, but its poor mechanical and thermal properties restrict its application [7]. As both cellulose and chitosan share similar chemical structures, it is possible to combine them, thereby complementing each other’s desirable properties to broaden their application [8]. This combination provides cellulose with new desirable properties, such as antimicrobial, antioxidant, and antiwrinkle effects. Additionally, cellulose/chitosan biocomposites offer enhanced water and metal ion adsorption, high porosity, excellent antistatic properties, improved mechanical characteristics, odor treatment properties, low cytocompatibility, and self-healing properties [6,9].

This review addresses gaps by providing a comprehensive analysis of cellulose–chitosan biocomposites, highlighting their synergistic properties, including enhanced mechanical strength, antimicrobial activity, and biodegradability. It examines their diverse applications in industries such as food packaging, medicine, and wastewater treatment, in line with the principles of green chemistry and the United Nations’ Sustainable Development Goals. This review also addresses important issues such as cost-effective production methods and future innovations, and proposes a roadmap for the introduction of these materials as sustainable alternatives to plastic.

## 2. Natural Sources for the Production of Biodegradable Plastics

### 2.1. Cellulose

**Figure 1:**
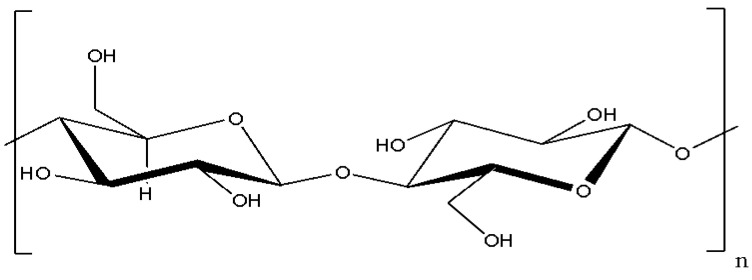
Structure of cellulose.

Cellulose is a linear, highly abundant biopolymer with the general formula of (C_6_H_10_O_5_)_n_. These glucose compounds are interconnected by β(1→4) glycosidic bonds, forming long linear chains, as shown in Figure 1. The linearity of the cellulose chains, which is facilitated by numerous hydrogen bonds between hydroxyl groups in neighboring chains, allows them to pack closely to each other. These hydrogen bonds create a rigid and stable crystal structure, significantly contributing to cellulose’s high tensile strength and hydrolysis resistance [10].

Cellulose is characterized by its white color, and lack of odor and taste. It is insoluble in water and most organic solvents due to its crystalline structure and extensive network of hydrogen bonds [11]. This insolubility makes processing difficult, but also makes cellulose stable in an aqueous environment, which is an important quality for plant cell walls and other natural structures [12].

Chemically, cellulose is relatively inert, especially in its natural crystalline form. It does not melt, but rather decomposes when heated to a temperature above 300 °C. This thermal stability is another result of its extensive hydrogen bonds and crystal structure [13]. However, cellulose can be chemically modified to increase its solubility and reactivity, making it a versatile material for various industrial applications. In an acidic environment, β(1→4) glycosidic bonds in cellulose can be hydrolyzed, splitting the polymer into shorter chains and glucose monomers [14].

Cellulose is obtained mainly from plants, where it is the main component of the cell wall. It can often be found with other organic compounds such as lignin or pectin. Cellulose plants include cotton, flax, hemp, coniferous and deciduous trees, fruits, nuts, and cereals. Moreover, cellulose sources can be bacteria such as Gram-negative, rod-shaped aerobic bacteria, which synthesize cellulose in high yields [15]. Most commonly, cellulose extracted from plant sources or microorganisms is converted into nanoforms such as nanofiber aerogels, hydrogels, nanoparticles, nanofilms, and nanocrystals with high surface-to-volume values and nanosize [16].

The efficiency of cellulose highly depends on the extraction method from its source and source features. Cellulose can be obtained from plant sources by chemical, physical, or enzymatic hydrolysis [16]. The list of cellulose sources and appropriate extraction methods are provided in Table 1.

### 2.2. Chitosan

**Figure 2:**
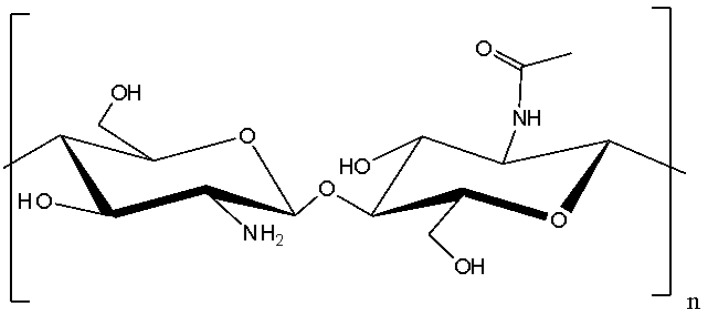
Structure of chitosan.

Chitosan is a biopolymer obtained from chitin, which is the second most common natural polysaccharide after cellulose [20]. Chitin is contained in the exoskeletons of crustaceans, such as crabs and shrimps, as well as in the cell walls of fungi and some insects [22,24]. Chitosan consists of β-(1-4)-linked D-glucosamine and N-acetyl-D-glucosamine and has a linear structure, as depicted in Figure 2 [25].

Chitosan is not a naturally found biopolymer, but can be obtained by performing deproteinization, demineralization, decolorization, and deacetylation of chitin. The deacetylation process involves enzymatic hydrolysis or chemical hydrolysis in a strongly alkaline solution. The degree of deacetylation depends on the hydrolysis conditions and chitin source, which significantly affects the properties of chitosan [20].

Chitosan has antimicrobial activity against a wide range of bacteria, fungi, and viruses. Moreover, chitosan is a biodegradable, biocompatible, nontoxic polysaccharide material that can form films chelating metal ions, which makes them widely used in industry and the biomedical field. The properties of chitosan mainly come from primary amino groups (pK_a_ = 6.3), which gives solubility in an acidic medium [26]. In acidic conditions, amino groups are protonated, and result in the formation of positively charged chitosan molecules. The positively charged chitosan molecule electrostatically interacts with negatively charged components, giving mucoadhesion properties. The solubility of chitosan in dilute acidic conditions makes chitosan a potential material for oral administration of anticancer drugs [27].

**Figure 3:**
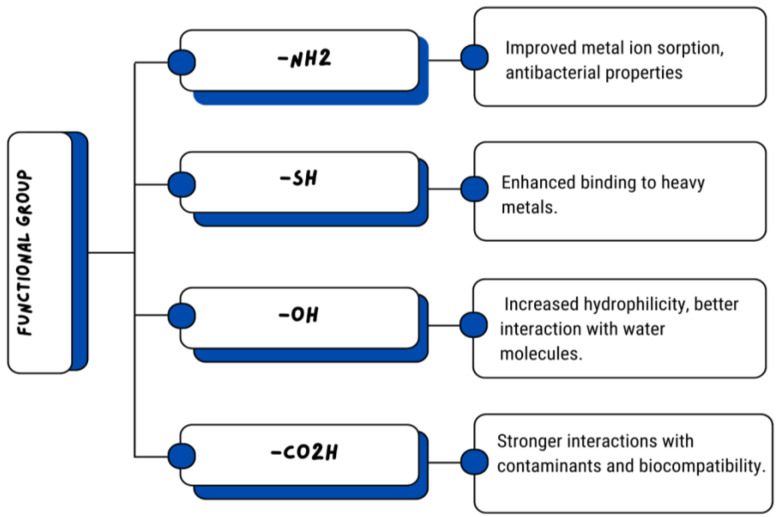
Functional group’s effect on properties.

Restriction on application of chitosan is based on its insolubility in water and in some organic solvents. To tackle this problem, chemical modification without changing distinctive properties is used. Both amino groups and hydroxyl groups in the backbone of chitosan provide reactive sites for chemical modifications, such as reductive amination, etherification, and esterification reactions. For example, modified chitosan derivatives such as thiolated chitosan and glycol chitosan have better bioactive properties [28]. The examples of improved properties by functional groups are illustrated in Figure 3.

Chitosan is an attractive material for biomedical applications because it exhibits the following properties: antifungal, anticancer, antidiabetic, antioxidant antitumor, antimicrobial, antibacterial, clotting time reduction, and analgesic properties [29,30,31].

## 3. Production of Bioplastics

**Figure 4:**
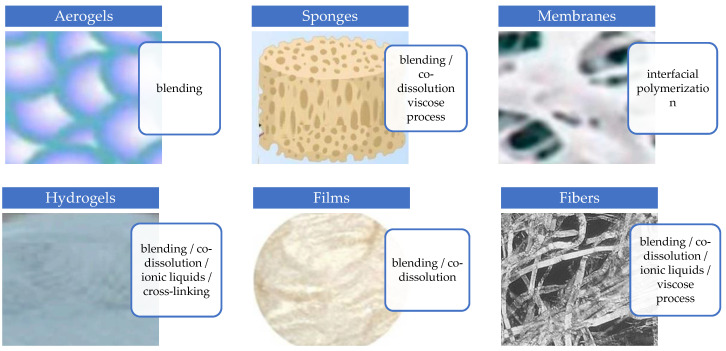
Preparation methods of cellulose/chitosan biocomposites.

The methods for preparing bioplastics can be classified into three categories: chemical, physical, and biological. The method for the preparation depends on the final structure/form of the cellulose/chitosan biocomposites and application, and the methods of preparation are given in Figure 4 [7].

### 3.1. Chemical Cross-Linking

One of the ways of preparing cellulose/chitosan hydrogel is cross-linking reactions, among which, chemical cross-linking is highly favorable as it offers better mechanical stability, but cytotoxicity for biomedical applications becomes a problem [32].

#### 3.1.1. Preparation of Reactive Cellulose

First of all, cellulose fibers are subjected to chemical treatments by periodate oxidation to convert 27.5% of hydroxyl groups into aldehyde groups. In an aqueous medium, 10.0 g of cellulose fibers, 6.6 g of sodium metaperiodate, and 14.5 g of sodium chloride are combined in 500 mL of deionized water in a glass beaker with an upper stirrer. The mixture is gently stirred at room temperature in the dark for 72 h. The modified cellulose with an aldehyde content is filtered and repeatedly washed with deionized water. Afterward, chlorite oxidation is conducted to convert 70% of aldehyde groups into carboxyl groups by treating 3 g of periodate-oxidized pulp with 1.62 g of sodium chlorite (80% pure), 5.85 g of sodium chloride, and 1.62 g of hydrogen peroxide (30 wt.% solution), and all dissolving in 200 mL of water. Subsequently, the reaction mixture is stirred at room temperature for 6 h, maintaining pH 5 by adding NaOH drops during the first 3 h. After completion, the fibers are extracted by adding ethanol (ethanol 2:1 to the reaction mixture), which facilitates coagulation and filtration. The product is washed twice with acetone and dried at room temperature [33].

#### 3.1.2. Preparation of Carboxymethylated Chitosan

Chitosan carboxymethylation starts by dissolving 1.35 g of sodium hydroxide in a propanol/water mixture (volume ratio 8:2). Then, 1 g of high-molecular-weight chitosan is added, stirred, and left to swell at room temperature for 1 h. Then, 1.5 g of chloroacetic acid is dissolved in 2 mL of propanol and then added to the chitosan suspension. Afterward, the mixture is reacted for 4 h at room temperature. The reaction is stopped by adding 50 mL of 70% ethanol and filtering through a nylon cloth. The remaining white solid material is washed four times with 80% ethanol and then washed with anhydrous ethanol. Finally, the mixture is dried in an oven at 50 °C to obtain carboxymethylated chitosan [33].

#### 3.1.3. Preparation of Cellulose–Chitosan Hydrogel

Reactive cellulose 1 wt.% solution is heated with 1 wt.% chitosan solution in three different ratios: 85:15, 75:25, and 65:35 (cellulose: chitosan, wt.%). The mixtures are mixed at 60 °C for 1 h. Two sets of samples are combined by magnetic stirring at 500 rpm for one minute and then left at room temperature for 4–6 h to form gels of cross-linked cellulose and chitosan [33].

### 3.2. Solvent Casting

Due to their similarity in structure, cellulose/chitosan biocomposites can be obtained through a blending process. Generally, there are two ways to obtain a blended cellulose/chitosan solution: dissolving each biomaterial separately in solvents or dissolving both biomaterials in one solvent. The second approach is challenging because of the difficulty of finding the right common solvent [7]. The blended cellulose/chitosan is obtained by the casting method.

Solvent casting is one of the most commonly used laboratory methods. This method involves three steps: solubilization, casting, and drying [34]. Using the casting methods, transparent bioplastic films with homogenous morphology, good mechanical strength, and a thickness of about 0.10 mm can be obtained [35]. This method includes expensive processing, which limits this method’s expansion to an industrial scale [34].

#### Preparation of Cellulose/Chitosan Biocomposites by Solvent Casting

The compounds 1 wt.% cellulose and 1 wt.% chitosan are added to a 60% LiBr solution and stirred at 300 rpm for 5 min. The solutions are mixed in mass ratios of cellulose and chitosan equal to 10:0, 9:1, 8:2, and 7:3. The suspensions are heated at 120 °C for 20 min until cellulose and chitosan become completely dissolved, and then stirred for another 10 min. The mixed solutions are transferred into a preheated glass mold (90 °C) to prevent thickening. The solution is thickened at a temperature of 70–80 °C, and then cooled to room temperature. The regenerated composite gels are washed with water. The composite gels are pressed at 2.5 kg for 30 min and then oven-dried at 105 ± 1 °C for 24 h to obtain films [36].

### 3.3. Electrospinning

Electrospun biomaterials have high porosity, large surface area, interconnectivity, hydrophilicity, antimicrobial, and good mechanical characteristics, making them especially attractive for tissue engineering and food industry applications [37,38].

The rigid structure of cellulose makes it difficult to obtain neat nanofibers. To solve this problem, co-spinning agents such as polyethylene oxide (PEO) are used to enhance electrospinnabilty, which is also biodegradable [39]. In addition, Song W. et al. proposed an innovative approach to recycle polystyrene waste into hyper-cross-linked polymers with broad functionalities, including potential for application in sustainable materials for biomedical and food technologies [40].

#### Preparation of Chitosan/Cellulose-Derived Nanofibers

CS/PEO solutions with a 1:1 mass ratio and a total polymer concentration of 2% (wt./vol.) are prepared in a 50% (vol./vol.) aqueous solution of acetic acid. The solution is homogenized for 5 min at 23,300 rpm using a homogenizer, and then centrifuged for 30 min at 5000 rpm to remove suspended particles and air bubbles. Then, various concentrations of cellulose (0.1, 0.5, 1, and 2% wt.) are added to the polymer solution. To avoid the formation of clusters, the powders are added carefully, and the polymer mixture should be vigorously stirred overnight for complete homogenization. Finally, the natural acacia extract is dissolved in a minimum amount of DMSO and added to the composition until a final concentration of 6 mg/mL is reached. The following optimized solution parameters are used: voltage 29 kV, flow rate 4 mL/h, cleaning frequency 90 s, head transverse speed 55 mm/s, speed rotation 100 rpm [39].

Table 2 shows the main advantages and disadvantages of the methods considered.

Thus, cellulose/chitosan biocomposites can be produced by various methods, including chemical cross-linking, solvent casting, and electrospinning. Each method has its own advantages and limitations related to mechanical strength, porosity, stability, and environmental friendliness. The choice of method depends on the end use of the material: for medical purposes, methods with high biocompatibility and porosity (e.g., electrospinning) are suitable, while for packaging or membranes, simple and scalable processes such as casting or chemical cross-linking are suitable.

## 4. Applications of Cellulose/Chitosan Bioplastics

The chitosan application is limited due to the brittleness, poor thermo-mechanical properties, and humidity sensitiveness, while cellulose application is limited because of the lack of antimicrobial properties. The cellulose/chitosan composite films demonstrate tensile strength improvements of 59% and a Young’s modulus increase by 42% [41]. Moreover, using the casting method, smooth and continuous surfaces of cellulose/chitosan bioplastic can be obtained [42].

Positively charged NH^3+^ in chitosan by interacting with negatively charged phosphoryl groups kill bacteria. Chitosan also prevents the proliferation of microorganisms by penetrating the nucleus, binding to DNA, and blocking RNA synthesis. In addition, chitosan can bind essential nutrients and metals, making them unavailable to microbes [41].

### 4.1. Cellulose–Chitosan Bioplastics in Food Packaging

Materials used for food packaging should protect food from moisture, oxygen, ultraviolet rays, and microorganisms in the environment [43]. Cellulose/chitosan bioplastics have a high potential for food packaging usage as well, as they possess antimicrobial properties, nontoxicity, low cost, a wide range of sources, mechanical strength, low density, and biodegradability. The cellulose–chitosan-based composite showed an oxygen transmission rate of 1.73 × 10^–11^ cm^3^·cm/cm^2^·s·Pa, and a water vapor transmission rate reduced to 2.24 × 10^–12^ g·cm/cm^2^·s·Pa, indicating that they can be good alternatives for conventional food packaging made of plastics [44].

Cellulose–chitosan-based materials for food packaging not only decrease the amount of plastics used in food packaging, but also improve the food quality by inhibiting microbial growth and extending the shelf life [45]. This is important as well, as 420,000 people die each year because of foodborne illnesses mainly caused by bacteria, parasites, and chemicals [46]. Furthermore, the increase in research on intelligent packaging from cellulose-based bioplastics makes cellulose–chitosan competitive alternatives for plastics. Cellulose/chitosan biocomposites are used for meat, milk, fruit, and vegetable food packaging [47]. One of the important uses of cellulose/chitosan bioplastics is in the food packaging of meat. Currently, cellulose–chitosan-based films used for meat packaging prevent meat spoilage under cold conditions by preventing an increase of the total volatile basic nitrogen (TVB-N), and offer an advantage over conventional packaging by offering effective food storage [48]. Chitosan films were also effective in reducing the growth of Pseudomonas and Enterobacteriaceae bacteria in meat [41]. Moreover, cellulose/chitosan biocomposites can be incorporated with indicators for real-time meat freshness monitoring of enclosed products with the naked eye. For example, Ezati et al. applied cellulose–chitosan/alizarin to detect beef spoilage, and the indicator changed color from brown to purple when the TVB-N amount in the beef increased [49]. Additionally, synergetic effects such as improved flexibility, good antioxidant activity, mechanical properties, and UV-light protection demonstrate cellulose–chitosan bioplastics as a promising material for food packaging [50].

Moreover, in recent years, cellulose/chitosan-derived edible coatings have been applied in the reduction of oil uptake and water loss in deep-fried foods. In addition to being applied to preformed solid sheets, the liquid form is applied to the food surface or between food components as barriers between food and fried oil [51].

### 4.2. Cellulose/Chitosan Bioplastics in Medicine

Cellulose–chitosan composite materials are widely used in medicine, primarily due to their biocompatibility, which ensures minimal adverse effects when interacting with human tissues. Their biodegradability allows them to decompose naturally in the body, reducing the need for surgical removal and minimizing long-term environmental impacts. Chitosan’s antimicrobial properties are beneficial for preventing infections, making these composites ideal for use in wound dressings and coatings for medical devices [28].

In addition, the excellent mechanical strength and flexibility of cellulose ensure structural integrity, and chitosan improves the functional properties of the composite, such as moisture retention and biological activity. This combination makes cellulose–chitosan composites suitable for a wide range of medical applications, including wound treatment, drug delivery, and tissue engineering. Their ability to promote cell adhesion and proliferation additionally supports tissue regeneration and healing processes [7]. The high porosity of biocomposites is critical for medical applications, as well as vascularization and cell migration depending on the property of the scaffolds. High surface area is important for cell attachment, while fiber directionality is essential for final scaffold mechanics [52]. In general, the synergy of cellulose and chitosan leads to the creation of modern materials that effectively solve various medical problems.

#### 4.2.1. Cellulose/Chitosan Bioplastics for Drug Delivery

Cellulose/chitosan biocomposites are suitable as drug delivery materials due to their hydrophobicity: active agents in the matrix can be released at the necessary rate without exceeding the toxic threshold dose [53]. This biocomposites possesses not only remarkable properties of commonly used polymer-based and silica aerogel drug delivery systems properties, such as high specific surface area and low thermal area, but also possesses biocompatibility and biodegradability, making them even more attractive than conventionally used drug delivery systems. The possibility of getting cellulose/chitosan biocomposites in different shapes and sizes broadens its application for drug delivery [54]. The cellulose/chitosan drug delivery systems can be used for both oral and nasal administration [55]. Moreover, cellulose–chitosan drug delivery systems also provide therapeutic effects. For instance, the vancomycin-loaded delivery system offered moisture balance at the wound, making the drug effective [54]. Other examples of other drugs used in matrix with cellulose/chitosan composite are provided in Table 3.

#### 4.2.2. Cellulose/Chitosan Bioplastics in Tissue Engineering

Hydrogels made of cellulose/chitosan biocomposites can mimic extracellular matrix, and assist cell promotion, cell differentiation, and cell adhesion more efficiently than synthetic polymers. Moreover, excellent mechanical properties, hydrophilicity, remarkable biocompatibility, and swelling properties make cellulose/chitosan a good candidate for tissue engineering [61].

- Cellulose/chitosan bioplastics for wound healing

Cellulose is widely used in wound dressings due to its high elasticity, excellent physical barrier against microbial pathogens, and, in particular, water-retaining properties, which are attributed to the abundance of hydroxyl functional groups [62]. It is well known that wounds heal faster in a moist environment, as it promotes a sufficient supply of growth factors. In addition, cellulose promotes the absorption of wound exudate and the removal of cell debris. However, cellulose itself does not have antibacterial and antifungal properties, making chitosan, with its inherent antibacterial and antifungal properties, a valuable addition to creating an ideal wound dressing. Traditional wound dressing materials have drawbacks such as instability and infection risk, while cellulose/chitosan-based wound dressing has antibacterial characteristics against *E. coli* and *S. aureus* [63]. Moreover, cytocompatibility, hemostatic capability, mechanical characteristics matching wound tissue, good bio-adhesiveness, and ultrafast healing over conventional wound dressings show high potential for cellulose/chitosan biomaterial [64,65]. Hydrogel made of cellulose/chitosan biocomposites can mimic extracellular matrix, and assist cell promotion, cell differentiation, and cell adhesion more efficiently than synthetic polymers. Moreover, excellent mechanical properties, hydrophilicity, remarkable biocompatibility, and swelling properties make cellulose/chitosan a good candidate for tissue engineering [61].

- Cellulose/chitosan bioplastics for the cartilage tissue engineering

Cartilage is avascular, so cartilage defects caused by trauma or aging are not repaired due to low cell density and lack of blood vessels in cartilage. Therefore, developing innovative tissue engineering techniques to repair cartilage defects and restore its function is of significant interest [66]. Cellulose/chitosan biocomposites have good shape recovery, similar stiffness properties as human cartilage, and high compressive strength, making it a promising material for cartilage repair [52]. In addition, chitosan interaction with chondrocyte cells leads to cartilage-like ECM production [67], and cellulose/chitosan promotes in vitro cartilage regeneration [68].

### 4.3. Cellulose/Chitosan Bioplastics for Wastewater Remediation

Cellulose–chitosan biomaterials have shown their potential in wastewater treatment due to their excellent adsorption properties, stability, and reusability. The combination of cellulose and chitosan is particularly effective because it uses the strengths of both materials. Cellulose and chitosan are attractive natural polymers for the manufacture of hydrogels since their numerous functional groups (hydroxyl and amino groups) provide numerous cross-linking sites during the preparation of hydrogels [69]. Overall, the development of cellulose–chitosan composite hydrogels offers a versatile, effective, and sustainable solution for the removal of various contaminants from wastewater, highlighting their potential for large-scale industrial and environmental applications [70].

Moreover, the addition of chitosan to cellulose hydrogels not only increases their metal adsorption capacity, but also increases the specific surface area and mechanical strength of the composite hydrogel compared to pure cellulose hydrogel. This makes the composite more efficient and durable for practical use [71].

#### 4.3.1. Cellulose/Chitosan Bioplastics for Removal of Heavy Metals

One notable study showed that a composite hydrogel consisting of 37% cellulose and 63% chitosan demonstrates an impressive adsorption capacity of 94.3 mg/g (1.49 mmol/g) for Cu^2^^+^ at 23 °C, pH 5, and an initial metal concentration of 1500 mg/L. This capacity was ten times higher than that of pure cellulose hydrogel. The composite hydrogel also demonstrated selective adsorption from a solution of mixed metals in the order Cu^2^+^^ > Zn^2^+^^ > Co^2^^+^. This innovative approach highlights the potential of using numerous and renewable natural polymers to create effective biosorbents to remove metal ions from water [72].

In another study, it was found that the vanadium adsorption capacity of cellulose/chitosan is 5.24 mg/g on the HS medium and 2.85 mg/g on the BCH medium at pH 4. This highlights the importance of the preparation method and the medium for optimizing the adsorption capabilities of cellulose-based materials [73].

Additionally, cellulose–chitosan biocomposites are used for the remediation of arsenic. However, the sorption potential is limited for arsenic, especially for As(III) compared to As(V). However, chemically modifying chitosan by adding high-affinity functional groups (such as −NH_2_, −SH, and −OH, etc.) improves its sorption capabilities. This enhancement happens due to increased interactions between arsenic and the high-affinity functional groups through mechanisms like electron donation, cation exchange, Lewis acid−base interaction, and surface complexation, leading to more effective arsenic removal [74].

Hydrogels made of cellulose/chitosan biocomposites can mimic extracellular matrix, and assist cell promotion, cell differentiation, and cell adhesion more efficiently than synthetic polymers.

#### 4.3.2. Cellulose/Chitosan Bioplastics for Removal of Anionic Dyes

The practical usefulness of these materials is demonstrated by repeated use of a specific cellulose–chitosan composite for the removal of anionic dyes. After ten adsorption–desorption cycles, its methyl orange adsorption capacity decreased from 95.83% to 94.57%, indicating that this material can be recovered and reused repeatedly, making it ideal for industrial wastewater treatment [75].

#### 4.3.3. Cellulose/Chitosan Bioplastics for Removal of Pharmaceuticals

In the fight against new pollutants such as pharmaceuticals, the prepared adsorbent magnetic hydrogel nanocomposite has demonstrated strong adsorption dynamics for propranolol hydrochloride, atenolol, and carbamazepine. At an optimal pH of 7.0, the removal efficiency can reach 98%. The hydrogel can be reused up to ten times while maintaining a removal efficiency of more than 80%. This efficiency has been well modeled by the Langmuir isotherm, which suggests that hydrogel is a promising and environmentally friendly option for removing beta-blockers and anticonvulsants from wastewater [76].

### 4.4. Cellulose/Chitosan Bioplastics for Stabilizing Pickering Emulsion

Emulsions are stabilized commonly by surfactants or polymers in different fields, including food and pharmaceutics. However, due to the lack of thermodynamic stability, considerable additive amounts, and toxicity of surfactants, they are not recommended to be used in these fields. The mechanism by which cellulose/chitosan stabilizes emulsions involves the interaction of electrostatic charges and steric hindrance between droplets. Along with this property, cellulose/chitosan biomaterial with superior stability, nontoxicity, good emulsifying capacity, low cost, high modulus, and ability to form a network at an oil–water interface makes this alternative one of the most promising Pickering emulsion stabilizer candidates [77,78].

**Figure 5:**
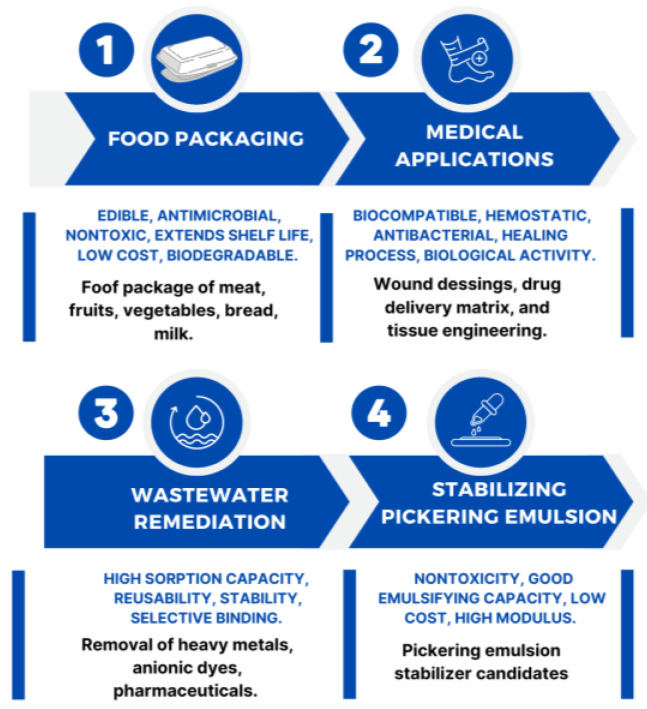
Applications of cellulose/chitosan biomaterial.

The summary of applications of cellulose/chitosan biocomposites is provided in Figure 5.

## 5. Conclusions

In conclusion, cellulose and chitosan-based biocomposites present a sustainable and effective alternative to conventional plastics, aligning with both green chemistry principles and the United Nations’ Sustainable Development Goals (SDGs). Their natural abundance, biodegradability, biocompatibility, and multifunctional properties make them suitable for various applications, including medicine, food packaging, and wastewater remediation. To fully realize their potential, further efforts are required to enhance the cost-effectiveness and scalability of their production processes, alongside the development of innovative applications. Advancements in regeneration techniques will extend the lifecycle of these biocomposites, reducing costs and environmental impacts. Achieving industrial-scale implementation will necessitate collaboration between industry, governments, non-governmental organizations, and academia. This cooperative approach will ensure that laboratory innovations are transformed into practical solutions, fostering a cleaner, safer, and more sustainable global environment, while addressing critical challenges like pollution, resource depletion, and public health.

## Figures and Tables

**Table 1:** Cellulose sources, cellulose content (%) or yield (g/L), and extraction method.

Source		Cellulose Content, % (Plant Sources) OR Yield, g/L (Bacterial Strain)	Extraction Method	Reference
Plants/trees	Cotton	94	acid hydrolysis	[17]
	Ramie	72	acid and alkali treatment	[15]
	Sisal	65	acid hydrolysis	[18]
	Sponge Gourd	60–63	acid hydrolysis	[19]
	Agava	60	acid hydrolysis	[20]
	Kenaf	55	acid hydrolysis	[21]
	Milkweed	51	acid hydrolysis	[22]
	Bamboo	44	acid hydrolysis, alkali bleaching process	[23]
Bacterial strain	*Gluconacetobacter* sp. RKY5 in glucose yeast extract broth	0.09–0.22	static cultivation	[15]
	*Acetobacter* sp. V6 in glycerol	4.98	agitated cultivation	
	Gluconacetobacter intermedius CIs26 in citrus waste media	7.2	static cultivation	
	Komagataeibacter hansenii C110 in stillgae	9.5	static cultivation	

**Table 2:** Advantages and disadvantages of the methods.

Method	Description	Advantages	Disadvantages
Chemical cross-linking	Chemical reaction between functional groups of cellulose and chitosan using cross-linking agents such as glutaraldehyde, carbodiimides (EDC/NHS), or epichlorohydrin.	- Strength and stability of the structure. - High water and chemical resistance. - Possibility of obtaining hydrogels and membranes with specified properties.	- Possible use of toxic reagents. - Need for thorough cleaning to remove reagent residues. - Limited biocompatibility if improperly cleaned.
Solvent casting	A mixture of cellulose and chitosan is dissolved in a common medium (e.g., acetic acid), then the mixture is precipitated or dried to form films, hydrogels, or membranes.	- Simplicity of the method. - Low cost of equipment. - Possibility of obtaining flexible and thin films or membranes. - Good compatibility of components.	- Limited mechanical strength. - Solvent dependent. - Difficulty in solvent removal. - Limited scalability.
Electrospinning	Formation of nanofibers from a mixture of cellulose and chitosan solutions under the action of a high-voltage electric field. Co-agents (e.g., polyethylene oxide) are often used to improve the process.	- High porosity and surface area. - Potential for tissue engineering, filtration, and sorption. - Possibility of regulating the diameter and structure of fibers.	- Difficulties in dissolving cellulose. - Dependence on the type of co-agents. - Need for precise control of parameters (voltage, solution concentration, and viscosity).

**Table 3:** Drugs applied with cellulose/chitosan matrix: application and release value.

Drug	Drug Application	Drug Release Value, %	Sources
Ciprofloxacin	urinary and respiratory tract infections	75	[56]
5-Fluorouracil	colorectal cancer treatment	86	[57]
Naringenin	hepatitis C, asthma, breast cancer	90	[58,59]
Quercetin	cardiovascular diseases, cancer, diabetes	92	[58,59]
Curcumin	metabolic syndrome, arthritis, hyperlipidemia	89	[58,60]

## Data Availability

Not applicable.
